# Verifying Proper Function of the Aerosol Evacuation System Prior to Sorting Potentially Infectious Samples

**DOI:** 10.1002/cpz1.70123

**Published:** 2025-03-21

**Authors:** Kristen M. Reifel, Avrill Aspland, Suat Dervish, Iyadh Douagi, Alyssa C. Fears, Evan R. Jellison, Cecily C. Midkiff, Taryn Mockus‐Daehn, Matilda J. Moström, Michael Solga, Brandon K. Swan, James Thomas, Stephen Perfetto

**Affiliations:** ^1^ Flow Cytometry Core, Vaccine Research Center, National Institute of Allergy and Infectious Diseases National Institutes of Health Bethesda Maryland; ^2^ School of Biomedical Engineering, Faculty of Engineering The University of Sydney Sydney Australia; ^3^ Westmead Research Hub The Westmead Institute for Medical Research Westmead New South Wales Australia; ^4^ Flow Cytometry Section, Research Technologies Branch, National Institute of Allergy and Infectious Diseases National Institutes of Health Bethesda Maryland; ^5^ Department of Microbiology and Immunology, Galveston National Laboratory University of Texas Medical Branch Galveston Texas; ^6^ Department of Immunology University of Connecticut School of Medicine Farmington Connecticut; ^7^ Division of Comparative Pathology Tulane National Primate Research Center New Orleans Louisiana; ^8^ Cellular Biomarkers, Biomarkers and Bioanalytical Platforms Precision Medicine, GSK Collegeville Pennsylvania; ^9^ Division of Immunology Tulane National Primate Research Center New Orleans Louisiana; ^10^ School of Medicine, Flow Cytometry Core University of Virginia Charlottesville Virginia; ^11^ National Biodefense Analysis and Countermeasures Center Frederick Maryland; ^12^ Florida Research & Innovation Center Cleveland Clinic Port Saint Lucie Florida

**Keywords:** aerosol containment, biosafety, cell sorting, flow cytometry, risk assessment, risk mitigation

## Abstract

High concentrations of aerosols can be generated within the sort collection area of cell sorters during instrument failures that cause the stream to deviate, such as a partial nozzle obstruction. Complete containment of these aerosol particles becomes essential for operator safety when working with potentially infectious or hazardous samples. Currently, aerosol containment is accomplished through the generation of continuous negative airflow within the sort collection area using an aerosol evacuation system, which can be enhanced by using primary containment devices such as biosafety cabinets. Unlike biosafety cabinets, many aerosol evacuation systems are not certified or tested on a regular basis after installation. Therefore, proper function of the system must be verified by the user prior to running hazardous samples to ensure that it is operational and provides sufficient protection for the operator. This protocol describes an updated procedure for verifying the containment and evacuation of aerosols generated when the stream is disrupted during an instrument failure. In this procedure, aerosols are generated to simulate a partial nozzle obstruction while running 1‐µm fluorescent beads. Air samples are collected just outside the sort collection area using a disposable impactor‐style aerosol sampler cassette and are examined for the presence of beads in an effort to detect aerosols. If no beads are present, aerosols were adequately contained and evacuated by the aerosol evacuation system. The presence of beads, however, indicates a potential failure of the aerosol evacuation system and/or other engineering controls that could result in the exposure of laboratory workers to any infectious or hazardous samples that are run through the instrument. © 2025 The Author(s). Current Protocols published by Wiley Periodicals LLC. This article has been contributed to by U.S. Government employees and their work is in the public domain in the USA.

**Basic Protocol**: Validation of the aerosol evacuation system using 1‐µm fluorescent beads and disposable aerosol sampler cassettes

**Support Protocol**: Preparation of 1‐µm fluorescent bead reference slide

## INTRODUCTION

Electrostatic droplet‐based sorting flow cytometers (cell sorters), where the stream is ejected at high velocity out of a nozzle, have been shown to produce high concentrations of aerosols within the sort collection area during partial nozzle obstructions or other malfunctions that disrupt the stream trajectory and cause the stream to impact a hard surface (Holmes, 2011; Holmes et al., 2014). The size and concentration of aerosol particles depend on the sheath pressure, with a greater potential for release of high concentrations (up to 25,000 particles/cm^3^) of small (1 to 5 µm) aerosol particles at high (≥70 psi) sheath pressures (Holmes, 2011). Aerosol particles in this size range present a higher risk because they can deposit in lung alveoli, are associated with increased infectivity of some organisms, and can remain airborne almost indefinitely (Cate et al., 1965; Day & Berendt, 1972; Vincent, 2005). Generation and release of aerosols by cell sorters poses a significant risk to laboratory workers because samples may contain live infectious agents and/or toxic, carcinogenic, or mutagenic dyes that could be encapsulated within aerosol particles (Schmid et al., 1997). To mitigate this risk, cell sorter manufacturers developed a customized aerosol evacuation system [also called aerosol management system (AMS) or aerosol management option (AMO)]. In this system, one or more hoses connect the sort collection area to the inlet of a primary containment device, such as a biosafety cabinet (BSC) or an external filtered vacuum unit, e.g., a PlumeSafe Whisper unit (Buffalo Filter). Typically, primary containment devices used for cell sorters are classified as Class II BSCs or enclosures that are functionally equivalent to Class II BSCs. However, any primary containment device can be used if it meets appropriate guidelines and passes appropriate tests. While the aerosol evacuation system is running, any aerosols generated by a stream deviation or similar malfunction (i.e., cell sorter‐derived aerosols) are contained within the enclosed sort collection area, and air containing these aerosols is evacuated through high efficiency particulate air (HEPA) or ultra‐low penetrating air (ULPA) filters. The aerosol evacuation system is considered a critical component of the engineering controls used to contain and evacuate aerosols generated by cell sorters (Holmes, 2011). These systems, however, are not generally certified or regularly tested after installation, and a procedure for validating proper containment of aerosols prior to cell sorter operation has historically not been available from manufacturers.

The updated protocol described here was developed by the International Society for Advancement of Cytometry (ISAC) Biosafety Committee to validate proper containment of aerosols by aerosol evacuation systems during cell sorter operation (Perfetto et al., 2019). In this protocol, 1‐µm fluorescent beads are run as a sample through the instrument while the stream is directed to impact a hard surface. This generates a continuous release of aerosols in the same manner as they would be produced during an instrument failure such as a partial nozzle obstruction. A disposable impactor‐style aerosol sampler cassette is then used to collect a defined volume of air just outside the sort collection area where most of the aerosols are generated. Aerosols that originated from the instrument and have escaped containment are identified by counting the number of beads on the impaction surface (i.e., cover slip) of the cassette using an epifluorescence microscope. “Test” samples are collected with the aerosol evacuation system on and set to the manufacturer recommended settings for cell sorting and all other manufacturer‐recommended controls in place. “Positive control” samples are collected in the same location(s) as the test samples but with the aerosol evacuation system turned off to allow aerosols to escape. Both test and positive control samples are collected while continuously generating the aerosol release. Any beads collected in test samples represent aerosol escape and a potential failure of the aerosol evacuation system. Preparation for this protocol involves acquiring the fluorescent beads, aerosol sampler cassettes, and vacuum pumps required to collect air samples, preparing a sample of fluorescent beads, performing the manufacturer‐recommended instrument startup including setup to generate an aerosol release, and setting up an epifluorescence microscope.

This article consists of a Basic Protocol outlining the steps to verify containment of aerosols by the aerosol evacuation system, instructions to prepare the 1‐µm fluorescent bead solution used in the Basic Protocol, and an optional Support Protocol to prepare a reference slide of 1‐µm fluorescent beads. In Basic Protocol, the 1‐µm fluorescent bead solution is run through the instrument while purposefully generating aerosols. Air samples are collected and examined for the presence of beads. The optional reference slide can be used to set up the microscope and troubleshoot issues with viewing and counting the fluorescent beads. The entire procedure can be completed in ∼60 to ∼90 min.

*CAUTION*: It is recommended to wear respiratory protection [e.g., N95, N100, or Powered Air Purifying Respirator (PAPR)] while generating and releasing aerosols as laboratory workers may be exposed to aerosols containing fluorescent beads and other chemicals and reagents.

## VALIDATION OF THE AEROSOL EVACUATION SYSTEM USING 1‐µM FLUORESCENT BEADS AND DISPOSABLE AEROSOL SAMPLER CASSETTES

In this protocol, proper containment and evacuation of cell sorter‐derived aerosols by the aerosol evacuation system are assessed by running a sample of 1‐µm fluorescent beads while purposefully generating aerosols, a proportion of which will contain beads. Air samples are then collected just outside the enclosed sort collection area in an effort to detect the beads. The fluorescent beads are used to identify cell sorter‐derived aerosols and distinguish them from background. At least one test sample and one positive control sample must be collected. Additional samples can be collected as desired (see Critical Parameters and Understanding Results). Note that some manufacturers have developed protocols to perform this test on specific cell sorter models. In these cases, follow the manufacturer's instructions for instrument settings and placement of cassettes.

### Materials

**Figure 1:**
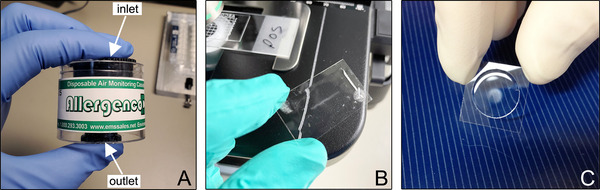
The Allergenco‐D as an example of an aerosol sampler cassette (**A**). Also shown are cover slips from a cassette with a slit nozzle and rectangular adhesive region (**B**) and a circular nozzle and round adhesive region (**C**). Both cover slips are from positive control samples showing visible dried sheath fluid where aerosols were deposited.

**Figure 2:**
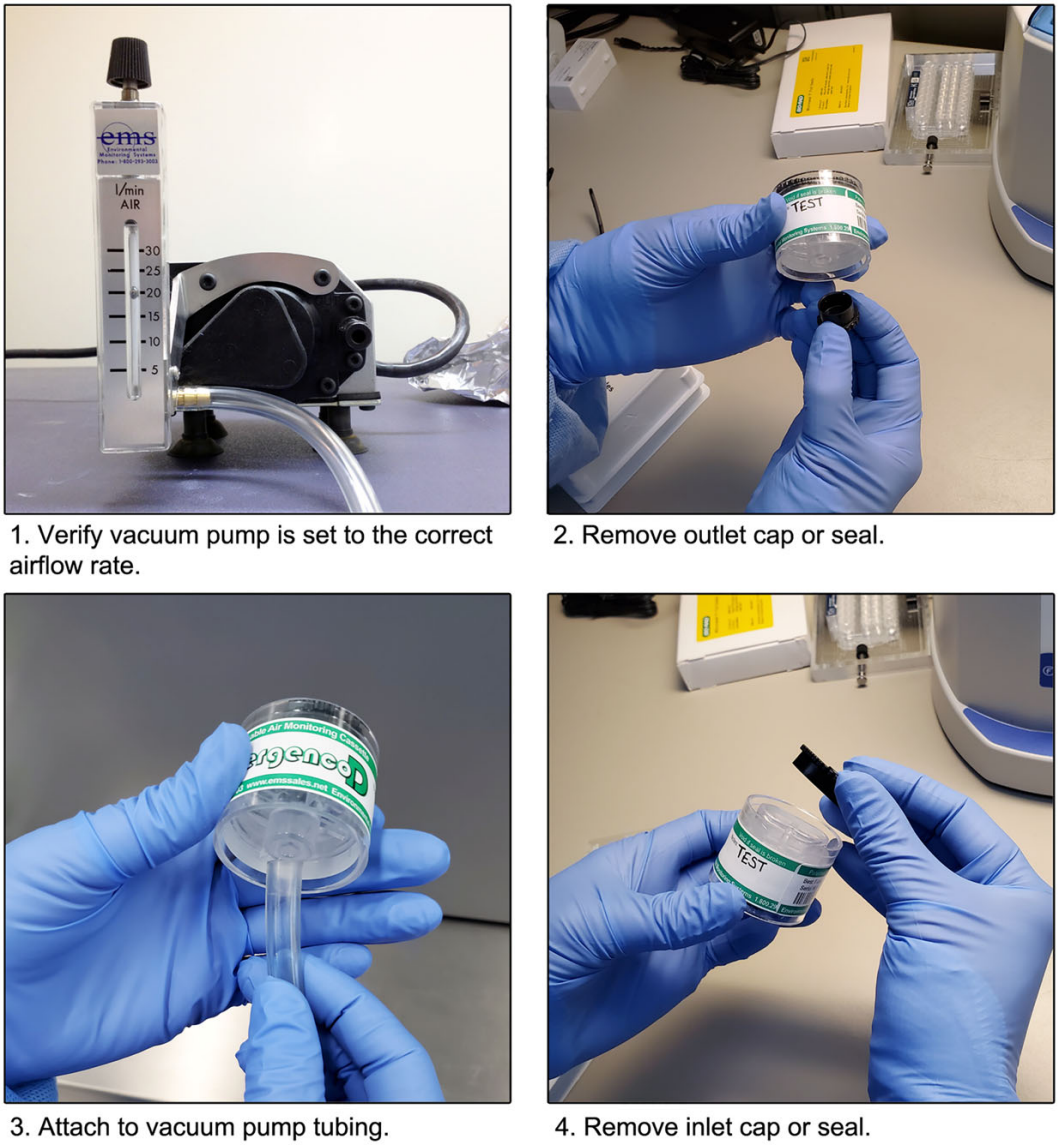
Preparing aerosol sampler cassettes for sample collection shown using the Allergenco‐D and corresponding vacuum pump.

**Figure 3:**
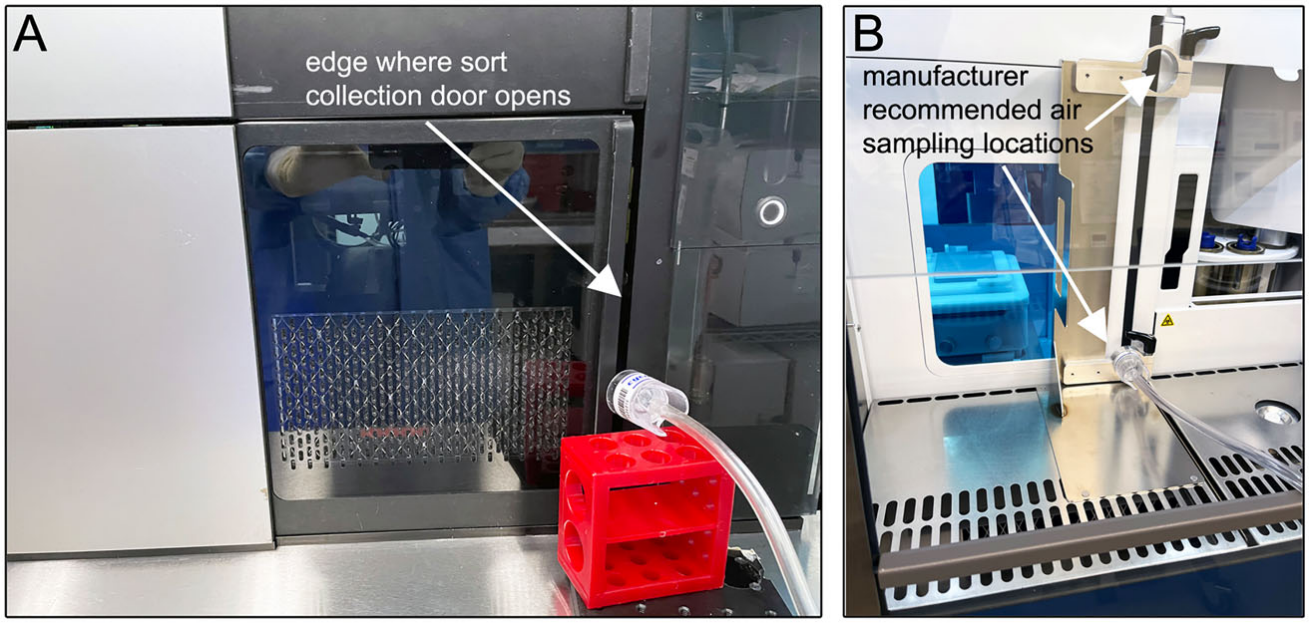
Examples of locations where aerosol sampler cassettes can be placed to collect test and positive control samples. Cassette placed near the opening edge of the sort collection door of a FACSymphony S6 cell sorter (BD Biosciences) (**A**). Cassette holders provided by the manufacturer, with one holding a cassette in one of the manufacturer‐recommended locations of a Bigfoot spectral cell sorter (Thermo Fisher Scientific) (**B**).

**Figure 4:**
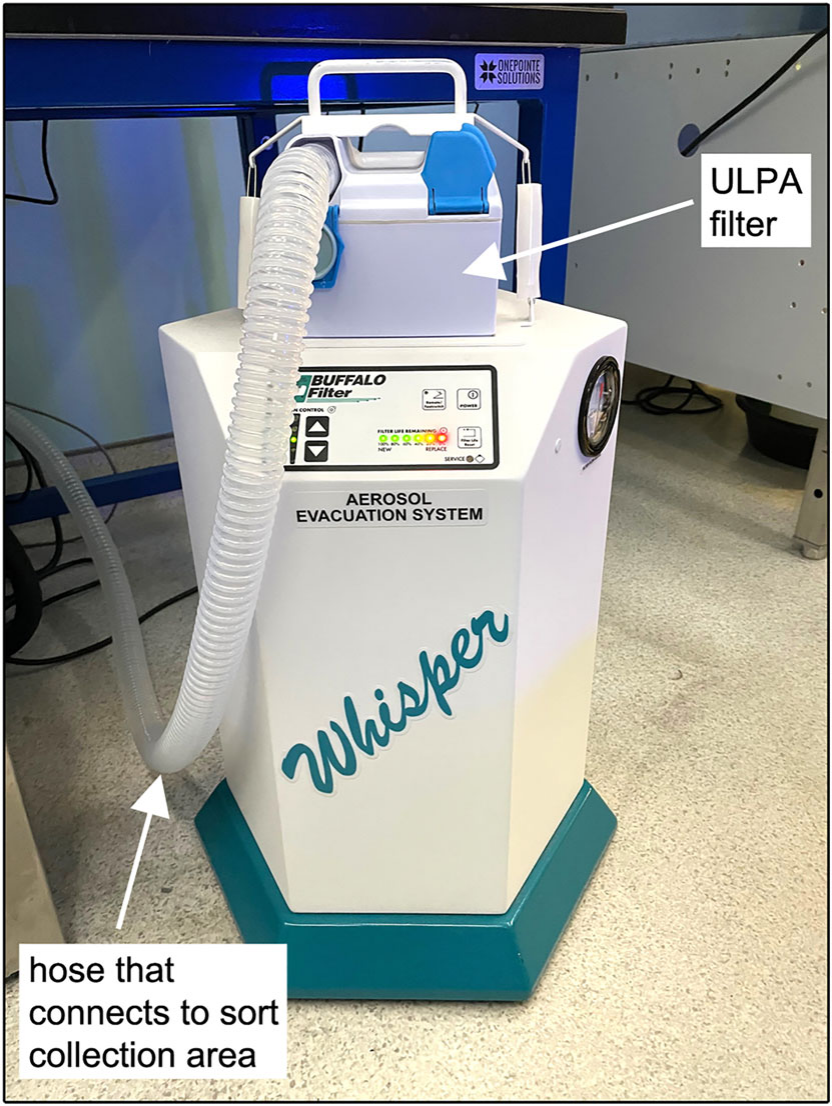
The Plumesafe Whisper unit as an example of an external aerosol evacuation system.

*NOTE*: This list includes reagents, materials, and equipment needed to perform the test. Materials needed to set up and run the cell sorter (e.g., sheath fluid, cleaning reagents, quality control beads, and alignment beads) are not included as these will vary between laboratories and instrument models. Reference laboratory‐ and manufacturer‐specific protocols for these items.

Disposable aerosol sampler cassette, with a cutoff diameter (d_50_) as close to 1 µm as possible, capable of sampling 200 L of air (Fig. 1; see Critical Parameters)
*Cyclex‐d cassettes (Environmental Monitoring Systems) were originally used but have been discontinued by the manufacturer. Other acceptable cassettes include Allergenco‐D (Environmental Monitoring Systems, cat. no. 120517), Air‐O‐Cell (Zefon International, cat. no. AOC010), and Via Cell (Zefon International, cat. no. VIA010). Non‐viable cassettes are ideal as this protocol does not attempt to collect living organisms*.
*These cassettes expire. They contain an adhesive that will dry out over time making them less efficient at capturing aerosols potentially affecting the sensitivity of the test. Use beyond the expiration date is not recommended*.
Vacuum pump with rotameter or other airflow indicator that can be set to the manufacturer‐recommended airflow for the cassette used
*Most cassette manufacturers sell a compatible vacuum pump with rotameter and attached tubing, such as the Basic Kit with IAQ Pump for the Allergenco‐D (Environmental Monitoring Systems, cat. no. 120790;* *Fig*. *2* *). However, any vacuum pump that can be adjusted to operate at the appropriate manufacturer‐recommended airflow rate and attached to the cassette is acceptable. Multiple vacuum pumps are required if multiple air samples will be collected at the same time*.
Plastic tubing
*Tubing is typically supplied with the vacuum pump (the Basic Kit mentioned above includes tubing) but may need to be purchased separately. Any tubing able to connect the vacuum pump to the cassette is acceptable*.
Stands or holders for cassettes
*Compatible stands can be purchased from cassette manufacturers. For example, the Basic Kit mentioned above includes a stand. Alternatively, common laboratory items, e.g., tube racks, retort stands, and tape can be used to hold cassettes near the point(s) of aerosol generation (* *Fig*. *3* *)*.
Timer, capable of measuring up to ∼20 min in 1 s intervals
Cell sorter
1‐µm Dragon Green bead (or other desired 1 µm fluorescent bead) solution (see recipe)
Aerosol evacuation system, either integrated into a primary containment device (Option A) or an external standalone system (Option B):
  Option A, primary containment device, such as a Class II BSC, with integrated aerosol evacuation system
*Custom primary containment devices supplied by instrument manufacturers are typically classified as or are functionally equivalent to Class II BSCs. However, any primary containment device that has passed appropriate tests and/or meets appropriate standards for your institution and global region (e.g., NSF/ANSI 49 standard in the US) can be used. The manufacturer can provide documentation of the classification of the primary containment device and the tests used to determine that classification. Institutional safety personnel should be consulted to determine whether the device meets the standards for your institution*.
*If the cell sorter is installed in a primary containment device without an integrated aerosol evacuation system, an external aerosol evacuation system can be used (Option B)*.
  Option B, external aerosol evacuation system, such as the Plumesafe Whisper unit (Fig. 4), for cell sorters installed on the benchtop
Disposable microscope slides
*Any standard disposable microscope slide is acceptable. Gridded slides are recommended (e.g., Electron Microscopy Sciences, cat. no. 63405‐01)*.
Microscopy immersion oil or mounting medium (e.g., immersion oil type A, Cargille, cat. no. 16482), optional
Reference bead that differs in size and/or peak fluorescence wavelength from Dragon Green beads (or other 1‐µm fluorescent bead), optional
*Examples of reference beads include: 1.0 to 1.4 µm high intensity Nile Red fluorescent particles (Spherotech, cat. no. FH‐1056‐2), and 6 µm Calibrite beads (BD Biosciences, cat no. 349502)*.
Spray bottle or other aerosolization device, optional
*Any bottle or device capable of spraying a small amount of bead solution is acceptable*.
Epifluorescence microscope
*Any model capable of viewing a standard microscope slide and generating light within the excitation and emission wavelengths appropriate for the fluorescent bead used during the test is acceptable (e.g., excitation and emission of 480 and 520 nm, respectively for Dragon Green beads). Note that light filters that do not exactly match the peak excitation and emission wavelengths of the beads may still be sufficient for visual detection of beads*.
Reference slide of 1‐µm Dragon Green or other desired 1‐µm fluorescent beads, optional (see Support Protocol for preparation instructions)

### Set up and preparation of aerosol sampler cassettes (5 min)

1 Ensure cassettes are within expiration. Label cassettes with the appropriate sample type (e.g., test, positive control, negative control) and sampling location if testing in multiple locations.
2 Verify the vacuum pump(s) is/are set to the manufacturer recommended airflow rate for the cassette model (typically 15 to 20 L/min) using attached rotameter or compatible flowrate meter (Fig. 2).

### Collect negative control sample (optional; 15 to 20 min)

This can be performed before or after instrument startup.

3 Remove the outlet cap or seal at the bottom of the cassette, if necessary, and connect to vacuum pump using appropriate tubing (Fig. 2).
4 Place cassette at or near the location where test and positive control samples will be collected using stands or holders as needed.
5 Remove the inlet cap or seal on the top of the cassette, if necessary (Fig. 2).
6 Set a timer to measure the desired sampling time.
  It is recommended to collect the same volume of air as in the test sample (200 L). Sampling time will vary depending on the manufacturer‐recommended airflow rate for the cassette used but typically ranges between 10 and 15 min. Sampling times can be rounded to the nearest 30 s if needed. For example, a sampling time of 13 min 30 s (13.5 min) can be used for cassettes that run at 15 L/min to collect ∼200 L of air.
7 Turn on vacuum pump and start the timer.
8 When the allotted sampling time has elapsed, turn off the vacuum pump.
9 Remove the cassette from the vacuum pump and replace any seals or caps.
10 Store cassette in the dark until analysis (step 48).

### Preparation of the cell sorter (5 to 10 min, not including cell sorter start‐up time)

**Figure 5:**
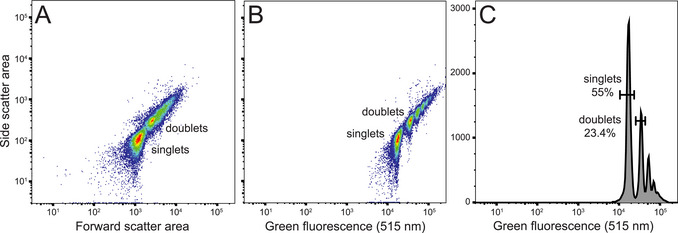
Examples of plots of the 1‐µm Dragon Green bead solution from data collected using a FACSymphony S6 cell sorter (trigger detector set to 515 nm) and plotted using FlowJo v10.10. All recorded events are shown (*n* = 32,330). The primary population of singlet beads is visible along with populations of doublets and other aggregates in all plots.

11 Start up and align the cell sorter following the manufacturer's instructions.
  - Set the sheath pressure to 70 psi, the maximum sheath pressure available for the instrument model, or the maximum pressure used for all workflows.
  - Alternatively, set the sheath pressure to the manufacturer‐recommended level if guidelines for the aerosol containment test are available for the cell sorter model used (see Critical Parameters).
  - In general, set the moveable parts of the cell sorter to match the settings or locations used for sorting. For example, set the aspirator drawer to its open or retracted position if using FACSAria, FACSymphony S6, or FACSMelody (BD Biosciences) cell sorters. The exact settings will depend on the design of the sort collection area and will vary with cell sorter models.
  Sorting is not required for this protocol. Setup for sorting (e.g., determination of drop frequency and drop delay) is optional.
12 It is recommended to remove items from the sort collection area that are not needed for the test to reduce clean‐up time. This could include collection device holders and deflection plates depending on the cell sorter model. Items such as absorbent toweling and/or containers to catch aerosolized or sprayed sheath fluid can be placed inside the sort collection area to help minimize cleanup.
13 Set the trigger detector to the fluorescence detector that most closely matches the peak fluorescence for the fluorescent bead used (e.g., 520 nm for Dragon Green beads) using the laser that most closely matches the peak excitation wavelength for the fluorescent bead used (e.g., 480 nm for Dragon Green beads).
  By using fluorescence as the trigger, total event rate can be used to monitor bead concentration as most events will be from beads and will exclude non‐fluorescent debris and noise.
14 Vortex or vigorously mix, load, and then run the 1‐µm fluorescent bead solution.
15 Adjust the trigger detector level and detector voltages as needed. Bead aggregates are common and are ok to keep in the range of detection (Fig. 5).
  If a large proportion of beads (>60%) appear as aggregates, vortex or sonicate the bead solution to break up the aggregates. Components to reduce the formation of aggregates (e.g., Tween 20) can also be added to the fluorescent bead solution (see Reagents and Solutions).

16 Adjust the sample pressure to achieve a total event rate of ∼50,000 events/s, or the maximum event rate possible for the cell sorter model. Alternatively, set the total event rate to the manufacturer‐recommended level if guidelines for the test are available for the cell sorter model used (see Critical Parameters).
17 Optional. Record a file of the bead solution.
  Sample flow can be stopped at this time and restarted just before collecting test samples (step 26).

### Collect test samples (15 to 20 min)

**Figure 6:**
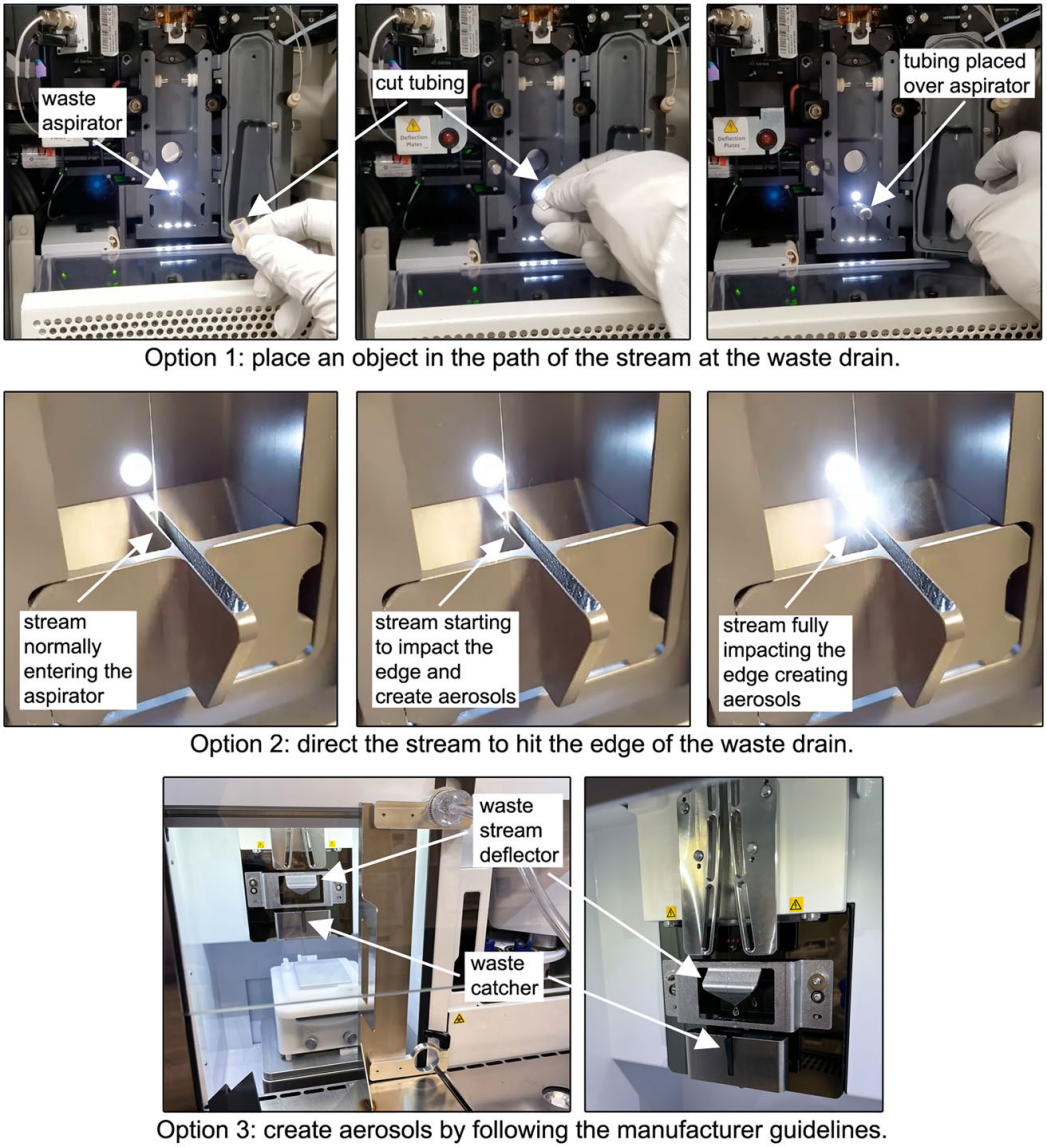
Methods to generate aerosols simulating a partial nozzle obstruction. Option 1: Place an object over the waste drain. Demonstrated using a FACSAria II cell sorter and a piece of cut tubing. Option 2: Direct the stream to impact the edge of the waste drain. Demonstrated using a FACSAria II cell sorter where the sort block assembly was tilted until the stream impacted the edge of the waste aspirator. Option 3: Create aerosols following manufacturer guidelines. The stream impacting the installed waste stream deflector on the Bigfoot spectral cell sorter is shown.

18 Remove the outlet cap or seal at the bottom of the cassette(s), if necessary, and connect to vacuum pump(s) using appropriate tubing (Fig. 2).
19 Place cassette just outside the sort collection area using appropriate stands or holders as needed.
  - If the instrument is installed inside a BSC (or other primary containment device), place the cassette inside the BSC unless it has been turned off.
  - Place additional cassettes in additional sampling locations as desired.
  - Alternatively, place cassettes(s) in location(s) recommended by the manufacturer if guidelines for the test are available for the cell sorter model used (see Critical Parameters).
  - See Figure 3 for examples of sampling locations.
20 If the instrument is installed within a BSC (or other primary containment device), verify the BSC is on and set to the manufacturer recommended settings used during sorting. Alternatively, the test can be performed with the BSC off if the aerosol evacuation system can be operated independently.
21 Verify the aerosol evacuation system is on and set to the manufacturer‐recommended settings used during sorting.
  The primary containment device and/or aerosol evacuation system may need to run for a period of time (e.g., 5 to 10 min) to establish proper airflow for containment. Check the manufacturer recommendations and allow warm‐up time if needed.
22 Verify all other engineering controls and components designed to contain and/or evacuate aerosols (e.g., sort collection area door, sample injection chamber door, vacuums at waste drains) are on and/or in place according to the manufacturer‐recommended settings for cell sorting.
23 Set a timer to measure the desired sampling time.
  200 L of air must be collected for the test sample. Sampling time will vary depending on the manufacturer‐recommended airflow rate for the cassette used but typically ranges between 10 and 15 min. Sampling times can be rounded to the nearest 30 s if needed. For example, a sampling time of 13 min 30 s (13.5 min) can be used for cassettes that run at 15 L/min to collect ∼200 L of air. Larger volumes of air can be collected if a higher level of sensitivity is desired.
24 Create a large release of aerosols by directing the stream to impact the edge of the waste drain or by placing an object in the path of the stream at the waste drain (e.g., cover the waste drain with tubing or parafilm). See Figure 6 for images showing methods to generate an aerosol release.
  CAUTION: To prevent false positives, do not turn off the aerosol evacuation system or open the sort collection area door while generating aerosols, especially if the 1‐µm fluorescent bead solution is running.
  CAUTION: Laboratory workers could be exposed to aerosols containing fluorescent beads and various chemicals. Consider using respiratory protection, especially if the instrument is not installed in a primary containment device.

25 Remove the inlet cap or seal on the top of the cassette(s), if necessary (Fig. 2).
26 Verify the 1‐µm fluorescent bead solution is running or restart sample flow if it was stopped earlier. Vortex or vigorously mix the bead solution if the sample has settled.
27 Turn on the vacuum pump(s) and start the timer.
28 When the allotted time has elapsed, turn off the vacuum pump(s).
29 Sample flow can be stopped at this time and restarted just before positive control samples are collected (step 37).
30 Remove the cassette(s) from the vacuum pump(s) and replace any seals or caps on the cassette(s) that were previously removed.
31 Store the cassette(s) in the dark until analysis (step 48).

### Collect positive control samples (15 to 20 min)

32 Remove the outlet cap at the bottom of the cassette(s), if necessary, and connect to vacuum pump(s) using appropriate tubing (Fig. 2).
33 Place cassette in the same location as the test sample (just outside the sort collection area) using appropriate stands or holders as needed. Place cassettes in additional sampling locations as desired. Alternatively, place cassette(s) in location(s) recommended by the manufacturer if guidelines for the test are available for the cell sorter model used (Fig. 3).
34 Set a timer to measure the desired sampling time.
  A sampling time of 2 min is recommended for the positive control sample. Shorter collection times can be used if very large numbers of beads and/or excessive liquid are collected over 2 min. Alternatively, sampling time can be lengthened to collect a higher number of beads if aggressive barriers and other engineering controls prevent most aerosols from escaping (see Understanding Results).
35 Remove the inlet cap or seal on the top of the cassette(s), if necessary (Fig. 2).
36 Turn off the aerosol evacuation system. It may also be necessary to partially or fully open doors around the sort collection area to allow aerosols to escape.
  CAUTION: Aerosols containing fluorescent beads and various chemicals can escape into the room. Respiratory protection is recommended for any laboratory workers present.
  Escaped aerosols could result in false positives if additional samples are collected after the positive control sample (see Critical Parameters). For instruments installed within a BSC (or other primary containment device), do not place hands inside the BSC once the aerosol evacuation system is off to reduce the likelihood of false positives outside the BSC.
37 Verify the 1‐µm fluorescent bead solution is running or restart sample flow if it was stopped earlier. Vortex or vigorously mix the bead solution if the sample has settled.
38 Turn on the vacuum pump(s) and start the timer.
39 When the allotted time has elapsed, turn off the vacuum pump(s).
40 Stop the sample flow.
41 Stop the generation of aerosols by redirecting the stream into the waste drain or removing the object in the path of the stream. The stream can also be stopped, if desired, to stop the generation of new aerosols.
42 Close any open barriers around the sort collection area.
43 Turn on the aerosol evacuation system. Run the aerosol evacuation system as recommended in the post clog procedure to evacuate aerosols from the sort collection area.
  If the instrument is installed inside a BSC (or other primary containment device), turn on the aerosol evacuation system before placing hands inside the BSC to reduce the likelihood of collecting false positives outside the BSC if any samples must be re‐collected.
44 Remove the cassette(s) from the vacuum pump(s) and replace any seals or caps.
45 Store the cassette(s) in the dark until analysis (step 48).
46 After aerosols have been evacuated from the sort collection area, thoroughly clean all surfaces that were exposed to aerosols, splashes, and/or spray containing the fluorescent bead solution.

### Prepare samples for microscopic analysis (5 to 10 min)

**Figure 7:**
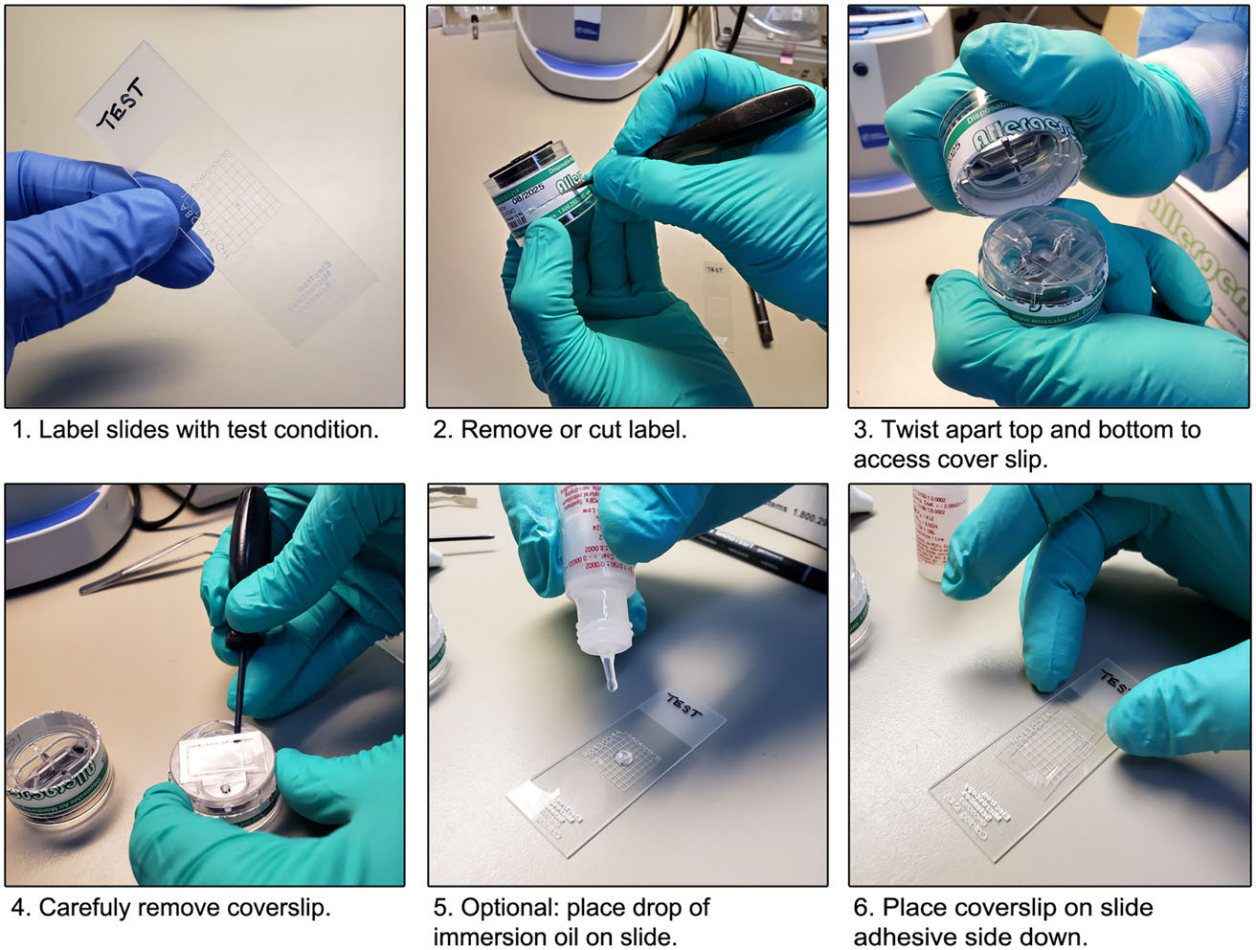
Preparing slides for microscopic analysis using the Allergenco‐D aerosol sampler cassette. A gridded slide was used where the grids are etched on the bottom of the slide. Optional use of immersion oil to help prevent the formation of bubbles is also shown.

47 Label microscope slides with the test condition and sample location from the corresponding cassettes if needed (Fig. 7).

48 Open the cassette by removing or cutting the label around the seam and twisting or pulling the two sides of the sampler apart (Fig. 7).
49 Carefully remove the cover slip using forceps and/or gloved hands (Fig. 7).
  CAUTION: Only touch the edges of the cover slip and do not disturb the adhesive coating.
  In some models (e.g., Allergenco‐D), small dots of adhesive are used to hold the cover slip in place inside the cassette. Use forceps (or other appropriate tool) to pry the cover slip up from the body of the cassette.
50 Optional. Place a drop of immersion oil or mounting medium onto a glass microscope slide (Fig. 7).
51 Optional. If using a reference bead, place a small drop onto the adhesive region of the cover slip. Alternatively, the bead solution can be diluted (1 drop in 1 to 2 ml of a diluent), added to a spray bottle, and a small amount sprayed onto the cover slip. A small amount can also be dropped or sprayed into the inlet of the cassette just before turning off the vacuum pump when collecting air samples (steps 8, 28, and 39 for negative control, test, and positive control samples, respectively).
  Reference beads may be useful for imaging slides that are not expected to contain any beads (e.g., test and negative control) but can be added to any slide as desired to help with microscope setup and focus. Examples of reference beads can be found in the Materials list above.
52 Place the cover slip, adhesive side down, onto the microscope slide (Fig. 7).
  If using gridded microscope slides, place the coverslip on the same side as the grid lines to ensure beads are in approximately the same focal plane as the grid lines.
53 Store slides in the dark until analysis. Slides will remain stable for 24 to 48 hr but should be viewed as soon as possible, ideally within the same day as collection.

### Count beads using an epifluorescence microscope (10 to 30 min)

**Figure 8:**
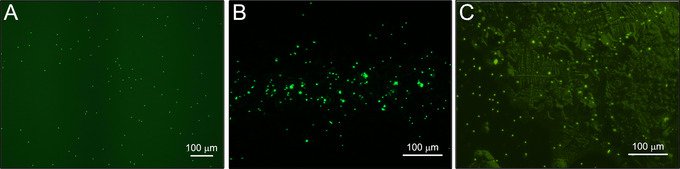
Microscope images of 1‐µm beads from positive control samples showing varying amounts of clumping and background (adhesive and dried salt from sheath fluid). Images taken using a ZOE Fluorescent Cell Imager (Bio‐Rad) (**A**), an EVOS FL Imaging System (Invitrogen) (**B**), and an Eclipse TS100‐F epifluorescence microscope (Nikon) (**C**).

54 Turn on and set up the epifluorescence microscope according to the manufacturer's instructions. The 10× and/or 20× objectives should be sufficient to view fluorescent beads. Higher magnification objectives are typically not needed.
55 Verify that the proper light filters are in place for the fluorescent bead used. For Dragon Green beads, a blue light source filter at or near 480 nm should be used for excitation, and a green filter at or near 520 nm should be used for viewing the beads. These filters are often combined within removable filter cubes equipped with mirrors.
56 Optional. View the reference slide at the desired magnification to set the light intensity, working distance, and focus. This should closely match the sample slides.
57 View the positive control slide(s).
  - If no reference slide was made, it is recommended to view this slide first before the test slide(s) to verify that the microscope is set up correctly, beads can be seen, and to orientate the observer to the bead size and focal plane.
  - Record whether the number of beads observed is greater or less than the tolerance level of 100 beads (or other tolerance level determined by the instrument model and sampling time, see Understanding Results).
  - See Figure 8 for microscope images of beads.
  It is not necessary to count all beads on the positive control slide. Verify that the slide contains at least 100 beads (or other tolerance level) by counting the number of beads in several fields of view until the tolerance level is met. If using a gridded microscope slide, the grids can be used as a guide to keep track of fields of view and maintain proper focus while scanning the slide.
  When large amounts of liquid aerosols are collected, beads may collect in clumps containing very high numbers where the liquid pools onto the coverslip adhesive. In these cases, it may be necessary to scan much of the adhesive region to find the clumps of beads, and once found, the number of beads in a field of view will be very high and can be estimated. Visible liquid on the cover slip also indicates that too many aerosols were collected. This does not negate the results of the test, but placement of the cassette and/or sample collection time can be adjusted to reduce the number of aerosols collected (see Understanding Results).

**Figure 9:**
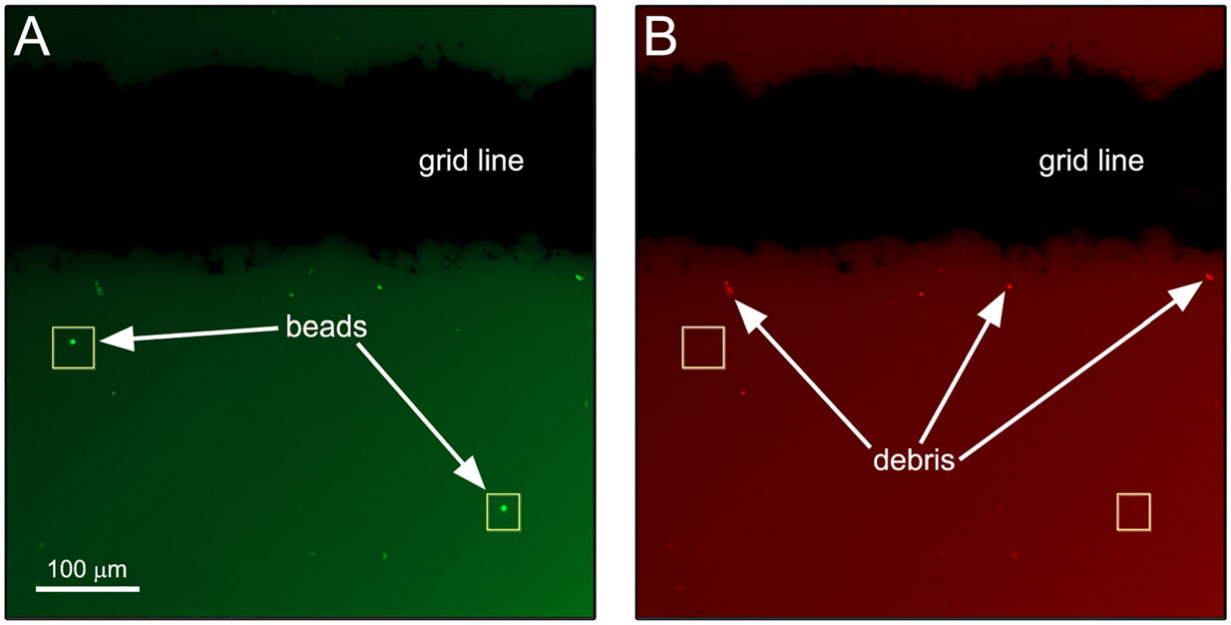
1‐µm Dragon Green beads and debris visualized using a gridded slide. The same field of view was imaged using a green filter (**A**) and a red filter (**B**) to help distinguish beads from debris. Images taken using an Axio Observer Z1/7 epifluorescence microscope (Zeiss).

58 If <100 beads (or other tolerance level) are observed on the positive control slide(s), check the cassettes, vacuum pumps, cell sorter setup, and fluorescent bead solution (see Troubleshooting). Re‐collect the positive control sample(s).
  For instruments installed within a BSC (or other primary containment device) that is running, this tolerance applies to positive control samples collected inside the BSC.
59 Optional. View the negative control slide. This slide will not contain any beads but will contain background debris from the ambient air and from other sources, such as immersion oil or mounting medium. Results from this slide can be recorded if desired but are not required to determine the test outcome.
60 View the test slide(s). Scan the entire adhesive region of the slide (Fig. 1) and record the total number of beads counted. See Figure 9 for images of beads along with background debris.
  To maximize sensitivity, scan the entire adhesive region. Although most aerosols are deposited near the central trace, beads may impact and stick at any point in the adhesive region. If using a gridded microscope slide, the grids can be used as a guide to keep track of the area scanned and maintain proper focus.

61 Optional. If available, toggle to a red emission filter (if using Dragon Green beads) to aid in distinguishing autofluorescent debris from beads (Fig. 9).
  Dragon Green beads are punctate spherical shapes and do not emit light within red wavelengths. Debris is likely to be visible under a red filter and will appear less uniform in shape.
62 If any beads are observed on the test slide(s) check that all components of the aerosol evacuation system (e.g., hoses, connections, HEPA or ULPA filter seals) and BSC or other primary containment device (if applicable) are set up and turned on according to the manufacturer's instructions and that all other controls are in place as recommended (see Troubleshooting). Re‐collect test sample(s).
  Before re‐collecting samples, cell sorter‐derived aerosols must be allowed to clear the room, and/or the BSC, to minimize the likelihood of false positives. Allow time for sufficient air exchanges to reduce aerosols in the room or the BSC (e.g., 30 to 60 min depending on airflow within the laboratory) and re‐collect samples (see Critical Parameters).
63 If at least 100 beads (or other tolerance level) are observed on the positive control slide(s) and zero beads are observed on the test slide(s), containment of aerosols by the aerosol evacuation system has been verified.
64 Beads observed on the repeat collection of the test slide(s) indicate a potential failure of the aerosol evacuation system. Containment of aerosols is NOT verified. Contact the manufacturer to perform diagnostic maintenance on the system. Re‐test after maintenance is completed.
65 For instruments installed within a BSC (or other primary containment device) that was running during the test, beads observed on the repeat collection of test and/or positive control slides collected outside the BSC indicate a failure to contain aerosols. Containment of aerosols is NOT verified. Contact the manufacturer to perform diagnostic maintenance on the system. Re‐test after maintenance is completed.

## PREPARATION OF 1‐µM FLUORESCENT BEAD REFERENCE SLIDE

It may be helpful to create a reference slide prepared using 1‐µm Dragon Green beads (or other desired 1‐µm fluorescent bead) (5 to 10 min). This slide can be used to set up the microscope, verify the optimal working distance and focal plane, adjust the light intensity to minimize autofluorescence of debris and background, and/or view how the beads appear when using different objectives. In this Support Protocol, preparation of a reference slide is described using a cover slip (i.e., the impaction surface) from a cassette of the same model that will be used for aerosol containment testing. Cover slips are coated on one side with an adhesive to prevent particle bounce. This adhesive can appear as a rough background and can introduce irregularities in the focal plane of beads when viewed using a microscope. A reference slide may help familiarize the observer's eye with bead size and brightness compared to this background and other debris that may be collected by the cassette.

### Materials

Aerosol sampler cassette
  The same model of cassette used in Basic Protocol should be used.
1‐µm Dragon Green beads (Bangs Laboratories, cat. no. FSDG004) or alternative 1‐µm fluorescent bead, if desired
  The same fluorescent bead that will be used in Basic Protocol should be used.
Disposable microscope slides
  Any standard disposable microscope slide is acceptable. Gridded slides are recommended (e.g., Electron Microscopy Sciences, cat. no. 63405‐01).
Spray bottle or other aerosolization device, optional
  Any bottle or device capable of spraying a small amount of bead solution is acceptable.
Reference bead that differs in size and/or peak fluorescence wavelength from Dragon Green beads (or alternative 1‐µm fluorescent bead), optional
  The same reference bead used in Basic Protocol should be used.
Microscopy immersion oil or mounting medium (e.g., immersion oil type A, Cargille, cat. no. 16482), optional

1 Open the cassette and remove the cover slip using forceps and/or gloved hands (Fig. 7).
  CAUTION: Only touch the edges of the cover slip and do not disturb the adhesive coating.
2 Place a small drop (maximum ∼2 µl) of concentrated 1‐µm fluorescent beads on the adhesive region of the cover slip. Avoid overfilling the slide. Alternatively, the concentrated bead solution can be diluted (1 drop in 1 to 2 ml of a diluent), added to a spray bottle, and a small amount sprayed onto the adhesive coating.
3 Optional. Place a small drop of the concentrated reference beads onto the adhesive region of the cover slip. Alternatively, the bead solution can be diluted (1 drop in 1 to 2 ml of a diluent), added to a spray bottle, and a small amount sprayed onto the cover slip.
4 Optional. Place a drop of immersion oil or mounting medium onto a microscope slide (Fig. 7).
5 Place the cover slip, adhesive side down, onto a microscope slide (Fig. 7). If using gridded slides, place the cover slip on the same side as the grids to ensure the beads and grid lines are in the same focal plane.
6 Store the prepared reference slide in the dark until ready for use.
7 As this prepared slide is not sealed, we recommend preparing a fresh reference slide each time the test is performed or every few days to weekly for laboratories who must perform frequent aerosol containment testing. Images from a prepared slide can be collected and saved as a visual reference.

## REAGENTS AND SOLUTIONS

### 1‐µm Dragon Green bead (or other desired fluorescent bead) solution (5 min)

The bead solution can be prepared using any 1‐µm fluorescent bead. The fluorescent bead should be bright enough to allow easy detection and separation from background and debris. Dragon Green beads are recommended because the excitation (480 nm, blue) and emission (520 nm, green) wavelengths are commonly used in most flow cytometry and microscopy laboratories, and they are very bright and easy to see at low magnification with an epifluorescence microscope. The bead sample should be prepared using the standard diluent used to prepare beads according to laboratory protocols. This is typically the same solution that is used as sheath fluid or a buffered salt solution such as phosphate‐buffered saline (PBS). Components to reduce clumping and/or keep beads in suspension (e.g., Tween 20) and increase shelf life (e.g., sodium azide) can be included if desired. Use a ratio of concentrated beads to diluent to achieve the desired event rate. This ratio will vary with factors such as instrument model, sheath pressure, and sample pressure. Prepare a final volume that is sufficient to complete all rounds of testing. Typically, ∼2 to ∼4 ml are needed depending on the sample flow rate and the time required to collect air samples. The bead solution can be stored for 1 to 2 days, but it is recommended to prepare a fresh bead solution each time aerosol containment testing is performed.

Example recipe for Dragon Green bead solution prepared for a FACSymphony S6 cell sorter run at 70 psi sheath pressure and ∼50,000 to ∼55,000 total events/s (5 min):

20 µl of 1‐µm Dragon Green beads (Bangs Laboratories, cat. no. FSDG004)
2 ml buffer (PBS with 0.1% sodium azide and 0.5% Tween 20)
Store up to 2 days at 4°C

Set an appropriate flow rate (typically 3 or 4) to achieve an event rate of 50,000 to 55,000 events/s.

## COMMENTARY

### Background Information

The ISAC Biosafety Committee set out to develop an improved test to verify proper function of the aerosol evacuation system that was relatively quick and easy to perform, was able to detect aerosols within the entire size range of interest (down to 1 µm) in a single assay, and with results that could be read the same day of scheduled sorting. To accomplish this, two major changes were made to previous testing protocols. First, 1‐µm fluorescent beads were used to label and identify cell sorter‐derived aerosols instead of T4 bacteriophage (Schmid et al., 1997) or Glo Germ fluorescent particles, which span a range of sizes (Holmes, 2011; Perfetto et al., 2003). Use of a fluorescent bead removes the need to prepare stock solutions of live organisms, which required preparations to begin up to a week before the test could be performed. The uniform 1‐µm fluorescent beads are also much easier than Glo Germ particles to identify and count microscopically and are easier to flush from the instrument once the test is complete. Second, a disposable impactor‐style aerosol sampler cassette was used to collect air samples instead of a reusable viable microbial sampler or relying on passive deposition onto Petri dishes. Cassettes are not reused, reducing the occurrence of false positives, are relatively inexpensive, are easy to use, and collect a wide range of aerosol sizes (determined by the d_50_ of the cassette, see Critical Parameters) including small aerosol particles that are of high concern for laboratory exposures (Holmes, 2011). Perfetto et al. (2019) demonstrated that the updated protocol using 1‐µm fluorescent beads and disposable cassettes was more efficient and reproducible than the T4 bacteriophage and Glo Germ protocols.

Perfetto et al. (2019) validated the protocol described here by comparing it to direct measurements of aerosol particle size (measured as aerodynamic diameter, d_a_) and concentration using an aerodynamic particle sizer (UV‐APS, TSI Incorporated). Aerosols were generated using a FACSAria II cell sorter and collected using Cyclex‐d cassettes (d_50_ of 1 µm). Internally fluorescent polystyrene beads in several sizes were used to label cell‐sorter derived aerosols. 1‐µm beads occupied aerosols within the size range of concern for cell sorter‐derived aerosols (1 to 5 µm) and were capable of being visualized and counted microscopically. Therefore, beads of this size were deemed a good surrogate for aerosols released by flow cytometers. Although the frequency of aerosols occupied by 1‐µm fluorescent beads was low (∼0.13%), the volume of air sampled (200 L) was sufficient to detect aerosols at concentrations down to ∼0.04 aerosol particles/cm^3^ with the instrument model and setup used (e.g., 50,000 events/s total event rate and 70 psi sheath pressure).

Other procedures to verify containment of aerosols may be available, and manufacturers may develop instrument‐specific procedures and/or monitoring devices in the future for this purpose. These procedures or devices could be used in lieu of the protocol described here to verify proper containment of aerosols if the chosen procedure has been validated against direct aerosol measurements (e.g., Perfetto et al., 2019) or has otherwise been shown to accurately detect cell sorter‐derived aerosols. It is critical, however, that proper containment of aerosols is verified periodically or in real‐time to prevent accidental aerosol release and keep laboratory workers safe. Performance of this test can be used as part of a risk mitigation plan to reduce risks of aerosol exposure identified in a risk assessment. We also recommend preparing Standard Operating Procedures (SOPs) for verifying proper function of these systems that are specific to the laboratory and/or instrument and contain specific tolerances, placement of aerosol sampler cassettes, and other important details. The protocol described here was broadly written so it could be adapted to a variety of instrument setups.

### Understanding Results

**Figure 10:**
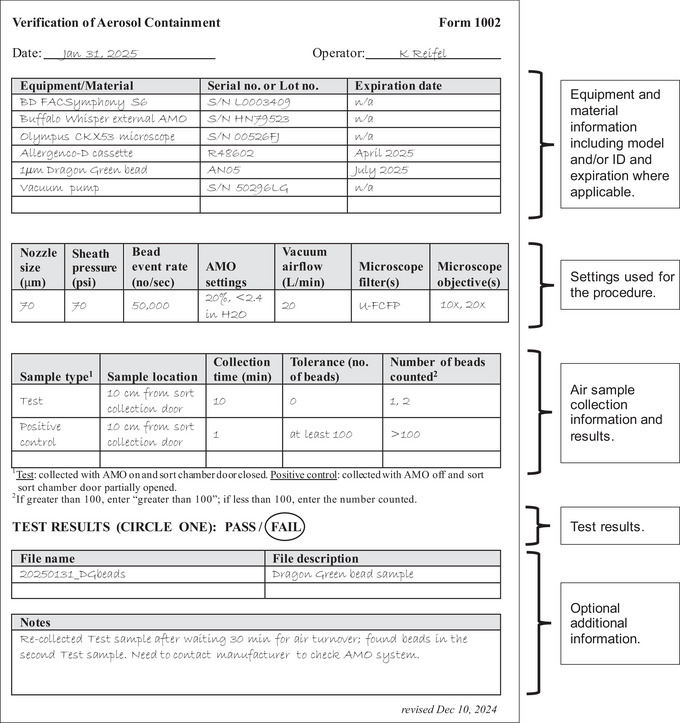
An example of a report sheet showing a suggested layout of information that should be recorded for each test.

Results of the test should be recorded in a report sheet and kept as a record of instrument performance. This sheet should include equipment information, materials and reagents, device settings, sample collection details, and test results. An example of a report sheet is shown in Figure 10.

#### Test sample

The purpose of the test sample is to challenge the system with a large and continuous aerosol release to determine whether the aerosol evacuation system is adequate to prevent aerosol escape. Test samples are collected with the aerosol evacuation system turned on and all other controls to contain aerosols in place at the manufacturer‐recommended settings for cell sorting (e.g., doors to the sort collection area and sample injection station closed, waste drain vacuums on). When the aerosol evacuation system is functioning properly, no beads will be collected in the test samples. Beads present in test samples indicate a potential failure of the aerosol evacuation system.

#### Positive control sample

The purpose of the positive control sample is to show that all aspects of the test are functioning as expected. Positive control samples are collected in the same location(s) as the test samples but with the aerosol evacuation system turned off to allow aerosols to escape. Positive control samples will contain a reproducible number of beads determined by the instrument model and exact setup and procedure used. In the original version of this procedure, Perfetto et al. (2019) routinely detected at least 250 beads in positive control samples collected for 2 min using a FACSAria II cell sorter running at 70 psi sheath pressure and a total event rate of 50,000 events/s. Thus, the tolerance for the positive control sample was set to ≥100 beads after collecting for 2 min. However, the number of beads collected in positive control samples could differ on other cell sorter models and with changes in sheath pressure, event rate, and/or sampling location. The positive control collection time and tolerance level can therefore be optimized for different cell sorter models. If very high numbers of beads are collected and/or visible liquid is observed in the cassette after 2 min, the collection time can be shortened. Alternatively, if <100 beads are consistently collected due to aggressive barriers and/or other engineering controls, the collection time can be lengthened and/or the tolerance can be reduced. It is not necessary to record the exact number of beads collected in positive control samples. The number of beads can be counted, or approximated, in several fields of view to verify the minimum tolerance was met, and the number can be recorded as greater than or less than the tolerance level (e.g., record “>100 beads” if at least 100 beads were observed, and the tolerance level is set to ≥100 beads).

#### Results for instruments installed within a primary containment device such as a BSC

For instruments installed within a BSC (or other primary containment device), test and positive control samples are collected inside the BSC so containment of aerosols within the sort collection area by the aerosol evacuation system can be tested. Airflow within the BSC can affect airflow from the sort collection area into the main body of the BSC potentially affecting the number of beads collected. Cassettes should be placed as close to the edge of the sort collection area door as possible to capture escaping aerosols. The collection time and/or tolerance level can also be adjusted for positive control samples as needed. Containment of aerosols by the BSC can be verified by collecting samples outside the BSC, if desired. However, this test should not be used as a replacement for BSC certification. Alternatively, the test can be performed with the BSC turned off if the aerosol evacuation system can be run independently.

#### Negative control sample

The purpose of the negative control sample is to collect and view any debris present in ambient air and/or other components (e.g., immersion oil). Viewing samples of background debris may aid in identifying and distinguishing beads collected in test samples. The slide made from this sample can be used as a reference when viewing the test and positive control slides. We recommend collecting negative control samples during establishment and optimization of the assay to characterize ambient room air and to aid laboratory workers to learn to identify debris. Negative control samples can be collected as often as desired if laboratory workers performing the test find them helpful, but they may not be needed once laboratory workers have gained experience viewing debris and beads. Note that beads should not be present in negative control samples. If beads are observed, refer to recommendations in “Preventing false positive results” (see Critical Parameters) including allowing time for air exchange in the laboratory after collecting positive control samples and instituting regular laboratory cleaning to prevent aerosolization of beads from surfaces or floors.

### Critical Parameters

#### Choice of aerosol sampler cassette

The original version of this protocol utilized the Cyclex‐d cassette (Perfetto et al., 2019). This non‐viable, single‐stage impactor‐style aerosol sampler cassette works by drawing in air that flows through a nozzle toward an impaction surface. The impaction surface consists of a coverslip with a sticky surface to prevent particle bounce. It uses inertia to collect a particular size range of aerosol particles that is determined by the d_50_, (the d_a_ at which the collection efficiency is 50%). Because this type of cassette is designed with a sharp collection efficiency curve, particles with a d_a_ less than d_50_ are not efficiently collected and pass through the device (Grinshpun et al., 2005; Lindsley et al., 2017). The Cyclex‐d has a d_50_ of 1 µm and can efficiently collect aerosols within the entire size range of interest for cell sorter‐derived aerosols (1 to 5 µm). However, the Cyclex‐d was recently discontinued by the manufacturer, and an exact replacement is currently not available. Several comparable cassettes are being evaluated by the ISAC Biosafety Committee. Our current recommendation is to choose a disposable cassette with a d_50_ within the size range of cell sorter derived aerosols and as close to 1 µm as possible. The cassette must also be able to sample the required volume of air for the test sample (200 L) without exceeding the manufacturer‐recommended sampling time. Collecting samples for extended periods of time could dry the adhesive coating, which could decrease the collection efficiency of the cassette reducing the sensitivity of the test. Non‐viable cassettes are ideal as this protocol does not attempt to capture living organisms, but viable cassettes are acceptable if they are disposable. Cassettes that meet these criteria include the Allergenco‐D (d_50_ = 1.7 µm), Air‐O‐Cell (d_50_ = 2.6 µm), and Via Cell (d_50_ = 1.56 µm). Note that in theory, cassettes with a larger d_50_ will be less sensitive as they will not efficiently collect the smallest cell sorter‐derived aerosols (aerosols below the d_50_).

#### Placement of aerosol sampler cassette(s)

The sort collection area is generally enclosed by a barrier with a door that can be opened to retrieve sorted samples. Aerosols produced in the sort collection area could escape along the edges where the door closes or seals. Thus, cassettes should be placed just outside the sort collection area at an edge to capture these escaping aerosol particles (Fig. 3). Positive control samples can be used to optimize the placement of cassettes during the initial implementation of this procedure. If little to no beads are observed on positive control slides, the cassettes may need to be placed closer to the edge of the door. For doors that seal when closed, the door may need to be at least partially opened to allow aerosols to escape. If very large numbers of beads and/or visible liquid is observed in positive control samples, too many aerosols have been collected. The cassette can be moved farther away from the sort collection area door and/or the air sampling time for the positive control sample can be reduced until a smaller number of aerosols is collected.

#### Sensitivity

High sheath pressures generate higher concentrations of smaller aerosol particles (Holmes, 2011), and the proportion of aerosols that contains a bead increases with increasing event rate (Perfetto et al., 2019). Thus, the test is recommended to be performed at a high sheath pressure and event rate to achieve the most sensitivity. Perfetto et al. (2019) recommend a sheath pressure and total event rate of 70 psi and ∼50,000 events/s, respectively. Using these settings on a FACSAria II cell sorter, they were able to detect cell sorter‐derived aerosols down to a concentration of ∼0.04 aerosols/cm^3^ when collecting 200 L of air. Although this lower limit of detection only applies under the original conditions where it was measured, it can be used as a guide to broadly estimate sensitivity under different conditions.

Some manufacturers have developed instrument‐specific protocols for this test that recommend particular sheath pressures and/or event rates (e.g., Thermo Fisher Scientific, 2024). In these cases, it is recommended to follow the manufacturer's instructions. Alternatively, the highest possible sheath pressure and event rate for the instrument model can be used if less than 70 psi and 50,000 events/s, respectively, or the highest sheath pressure used for all laboratory protocols can be used. Results of the test are valid for all sheath pressures equal to or less than the tested sheath pressure. If higher sheath pressures will be used, the test must be repeated at the maximum sheath pressure to verify the higher aerosol load is contained by the aerosol evacuation system and engineering controls. Nozzle size is likely to have a smaller effect on the concentration and/or size distribution of aerosols, but these effects have not been characterized. We suggest using the same nozzle size typically used for cell sorting to best approximate aerosols that would be released during a stream deviation. In general, sensitivity can be increased by collecting a larger volume of air in the test sample (without exceeding the manufacturer's recommendations), which decreases the detection limit and, to a lesser extent, by running the bead sample at a higher event rate to increase the proportion of aerosols that contains a bead. Note that collecting smaller volumes of air (e.g., shortening collection time of the test sample) will increase the detection limit and reduce the sensitivity of the test.

#### Preventing false positive results

Because the sensitivity of the test is high (one bead in the test sample results in a failed test), it is important to take measures to prevent false positives. When collecting the positive control sample, potentially high concentrations of aerosols containing fluorescent beads are released into the room and/or into the BSC (or other primary containment device). It is therefore recommended to collect these samples last. For instruments installed inside a BSC, any airflow disturbance could result in escape of aerosols containing beads into the room especially when the aerosol evacuation system is off. Thus, all manipulations inside the BSC should be performed before turning off the aerosol evacuation system. If samples must be collected after the positive control samples, wait an appropriate amount of time (e.g., 30 to 60 min depending on the air turnover rate in the room and/or the BSC) before attempting sample collection to allow time for sufficient air exchanges to remove beads from the ambient air.

Many types of fluorescent beads are regularly used in flow cytometry laboratories. These beads could end up on various surfaces through spills, splashes, or other sample manipulations and have occasionally been observed in test samples. It is therefore recommended to regularly clean laboratory floors and other surfaces to prevent these beads from being aerosolized and collected potentially resulting in false positive results. Surfaces exposed to aerosols, splashes, or spray should also be cleaned after performing aerosol containment testing to remove fluorescent beads.

#### Testing frequency

At a minimum, proper function of the aerosol evacuation system should be verified following instrument installation, service or maintenance involving any component of the sort collection area or aerosol evacuation system, changeout of the HEPA or ULPA filters on the external aerosol evacuation vacuum unit or the BSC (or other primary containment device), and if the instrument is removed from and replaced within the BSC. Aerosol containment can also be verified on a regular basis (e.g., annually, quarterly, monthly, or weekly) and/or just prior to sorting. The required frequency and timing of testing depends on the risk level of the sample(s) and the risk tolerance of the laboratory/institution and should be determined through a risk assessment performed in collaboration with institutional biosafety personnel.

### Other Applications

This protocol can be modified to test other areas of concern on cell sorters, operation of cell sorters under different conditions (e.g., without an aerosol evacuation system), and areas of concern on other instruments that potentially release aerosols (e.g., flow cytometer analyzers). Aspland et al. (2021), for example, found moderate to high concentrations of aerosols at the waste tanks of analyzer instruments by running 1‐µm fluorescent beads and collecting air samples using Cyclex‐d cassettes near the waste tank lid. In addition, some of our laboratories have discovered faulty ULPA filters in external aerosol evacuation systems by consistently observing low numbers of beads in test samples and low to moderate numbers of beads in samples collected where air is exhausted from the vacuum unit. This procedure can also be used to determine the amount of time needed to evacuate aerosols from the sort collection area by collecting air samples upon opening the sort collection area door after waiting a defined amount of time.

### Troubleshooting

See Table 1 for a troubleshooting guide with suggestions and recommendations for addressing potential issues and unexpected results that can occur when implementing this procedure.

**Table 1:** Troubleshooting Guide for Addressing Potential Issues and Unexpected Results*^a^*

Problem	Possible cause	Solution
Beads present on negative control slide	Environmental debris mistaken as fluorescent beads	Compare to positive control slide and/or reference slide to verify that fluorescent particles are indeed beads
Beads present on negative control and/or test slide	False positive due to contamination	Wait ∼30‐60 min (depending on air turnover rate) for aerosolized beads to be removed from the air via ventilation; thoroughly clean all laboratory surfaces; change gloves to prevent transfer of beads to cover slips
Beads present on test slide	Aerosol evacuation system not setup/connected correctly and/or airflow was not established	Check the connections, seals, and hoses, and verify the system is set to the manufacturer‐recommended settings for cell sorting; allow a warmup time for proper airflow to be established (e.g., 5‐10 min but see manufacturer recommendations)
No beads present on positive control slide	Aerosol evacuation system is running	Ensure the aerosol evacuation system has been turned off; for some instruments, it may be necessary to wait a few minutes for the system to completely stop running after powering off
No beads present on positive control slide	Fluorescent bead solution is not running or has settled	Ensure the fluorescent bead sample is running before collecting air samples; vigorously mix or vortex the fluorescent bead solution before running; the bead sample may need to be mixed periodically during the test procedure; adding a detergent (e.g., TWEEN 20) to break surface tension can help keep beads in suspension
No beads visible on positive control slide	Epifluorescence microscope not set correctly or not compatible with beads	Ensure the epifluorescence microscope is equipped with the appropriate light filter(s) that match the absorption and emission peaks of the fluorescent bead(s) being used, and that they are in place
Beads counted on the positive control slide are below the threshold	Aerosol sampler cassette error	Ensure the cassette is placed in an optimized location, is not expired, is properly attached to the vacuum pump, the protective coverings/caps are removed, and the vacuum pump is turned on and set to the appropriate flow rate
Beads counted on the positive control slide are below the threshold	Incorrect sampling time and/or threshold	Set a longer sampling time or a new threshold for the positive control sample
Number of beads on test slide comparable to positive control slide	Aerosol evacuation system is not functioning	Ensure the aerosol evacuation system is turned on; contact the manufacturer if beads are still present on the test slide
Unable to remove cover slip from cassette	Cover slip is attached to the body of the cassette	Find the areas where the cover slip is attached by the adhesive; using an appropriate tool (e.g., forceps), carefully pry up each attached area
Unable to distinguish beads from autofluorescent debris and background	Light intensity is too high	View a reference or positive control slide; find a field of view that contains fluorescent beads; reduce the light intensity until the background appears darker but the beads are still clearly visible
Unable to view beads or debris on slides	Light source has not reached full brightness	Turn on the microscope light source and allow an appropriate warm‐up time, depending on the type of light source, before attempting to view slides
Unable to focus on beads/debris	Incorrect microscope settings	Refer to the manufacturer's instructions to verify that the proper settings are in use to optimize resolution and contrast
Unable to focus on beads/debris	Beads and/or debris may be in different planes of focus	Adjust the focal plane as needed while viewing different areas of the slide; some beads may remain out of focus due to limitations in microscope working distance, however, out‐of‐focus beads produce round halos, which may assist in distinguishing beads from debris; change the plane of focus while viewing a positive control slide to verify this effect
Excessive debris on slides	Slides are dirty	Clean slides with lint‐free toweling or lens cleaner wipes before use

^*a*^ A “live” version of this table will be maintained on the ISAC Biosafety Committee webpage.

### Time Considerations

This protocol was designed to be completed in a relatively short amount of time so it can be performed on the same day as sorting. With practice, the entire protocol can be completed in ∼60 min, but the exact time depends on details, such as the air sample collection time and the number of air samples collected. Note this time estimate does not include instrument startup and optional steps (e.g., preparation of the reference slide, collection of the negative control sample). Some instruments may also require extended flushing or additional cleaning steps to remove residual 1‐µm fluorescent beads.

We recommend initial optimization for laboratories who have never attempted this procedure and after installation of new instrument models that have never been tested. The goal of this optimization is to determine the best way to create a continuous aerosol release and to optimize placement of cassettes, collection time for the positive control sample, and other protocol details that change with the specific instrument setup. Start with the basic version of the test, and include optional components (e.g., reference beads) as needed to address issues. Laboratories should allow half a day to a full day for testing to optimize the protocol. They should also work with the instrument manufacturer to determine optimal settings based on specific details of the instrument model. Once optimized, protocol details such as the bead solution recipe, instrument settings needed to achieve the desired event rate, collection time for the positive control sample, exact placement of cassettes, optimal microscope settings, and post‐procedure cleaning steps can be included in laboratory‐specific SOPs or other guidance documents.

### Author Contributions

**Kristen Reifel**: Conceptualization; project administration; writing—original draft. **Avrill Aspland**: Conceptualization; writing—review and editing. **Suat Dervish**: Writing—review and editing. **Iyadh Douagi**: Conceptualization; writing—review and editing. **Alyssa Fears**: Writing—review and editing. **Evan Jellison**: Methodology; writing—review and editing. **Cecily Midkiff**: Writing—review and editing. **Taryn Mockus‐Daehn**: Writing—review and editing. **Matilda J. Moström**: Writing—review and editing. **Michael Solga**: Writing—review and editing. **Brandon Swan**: Conceptualization; writing—review and editing. **James Thomas**: Writing—review and editing. **Stephen Perfetto**: Writing—review and editing.

### Conflict of Interest

The authors have no conflicts of interest to declare.

## Data Availability

Data sharing not applicable to this article as no datasets were analyzed during the current study.
